# Supplementary material for: ALKBH5 controls the meiosis-coupled mRNA clearance in oocytes by removing the N 6-methyladenosine methylation
Source: Nat Commun. 2023 Oct 17;14:6532. doi: 10.1038/s41467-023-42302-6 (PMC10582257; doi:10.1038/s41467-023-42302-6)
Supplement: Supplementary file 1 — Supplementary Information [file 41467_2023_42302_MOESM1_ESM.pdf]

Supplementary Information

ALKBH5 controls the meiosis-coupled mRNA clearance in oocytes by removing the N^6-methyladenosine methylation

Long Bai[1, 2, #, *], Yu Xiang[1, 2, #], Minyue Tang[1, 2, #], Shuangying Liu[1, 2], Qingqing Chen[1, 2], Qichao Chen[1, 2], Min Zhang[1, 2], Shan Wan[1, 2], Yimiao Sang[1, 2], Qingfang Li[1, 2], Sisi Wang[1, 2], Zhekun Li[1, 2], Yang Song[1, 2], Xiaoling Hu[1, 2], Luna Mao[1, 2], Guofang Feng[1, 2], Long Cui[1, 2], Yinghui Ye[1, 2], Yimin Zhu[1, 2, *]

[1] Department of Reproductive Endocrinology, Women's Hospital, School of Medicine, Zhejiang University, Hangzhou, Zhejiang 310002, China.

[2] Key Laboratory of Reproductive Genetics (Ministry of Education) and Women's Reproductive Health Laboratory of Zhejiang Province, Women's Hospital, Zhejiang University School of Medicine, Hangzhou, Zhejiang 310002, China.

This combined PDF file contains the following Supplementary figures:

Supplementary Figure 1. Dynamic ALKBH5 distributions during meiosis in mouse oocytes.

Supplementary Figure 2. Genetic knockout of *Alkbh5* and analysis of *Alkbh5*$^{-/-}$ female phonotype.

Supplementary Figure 3. *Alkbh5* depletion impairs cell cycle in meiosis and mitosis.

Supplementary Figure 4. Disrupted transcriptomes in oocyte maturation upon *Alkbh5* knockout.

Supplementary Figure 5. Characteristics of m^{6}A modifications in *Alkbh5*$^{-/-}$ oocytes.

Supplementary Figure 6. ALKBH5 also functions before the occurrence of maternal RNA decay.

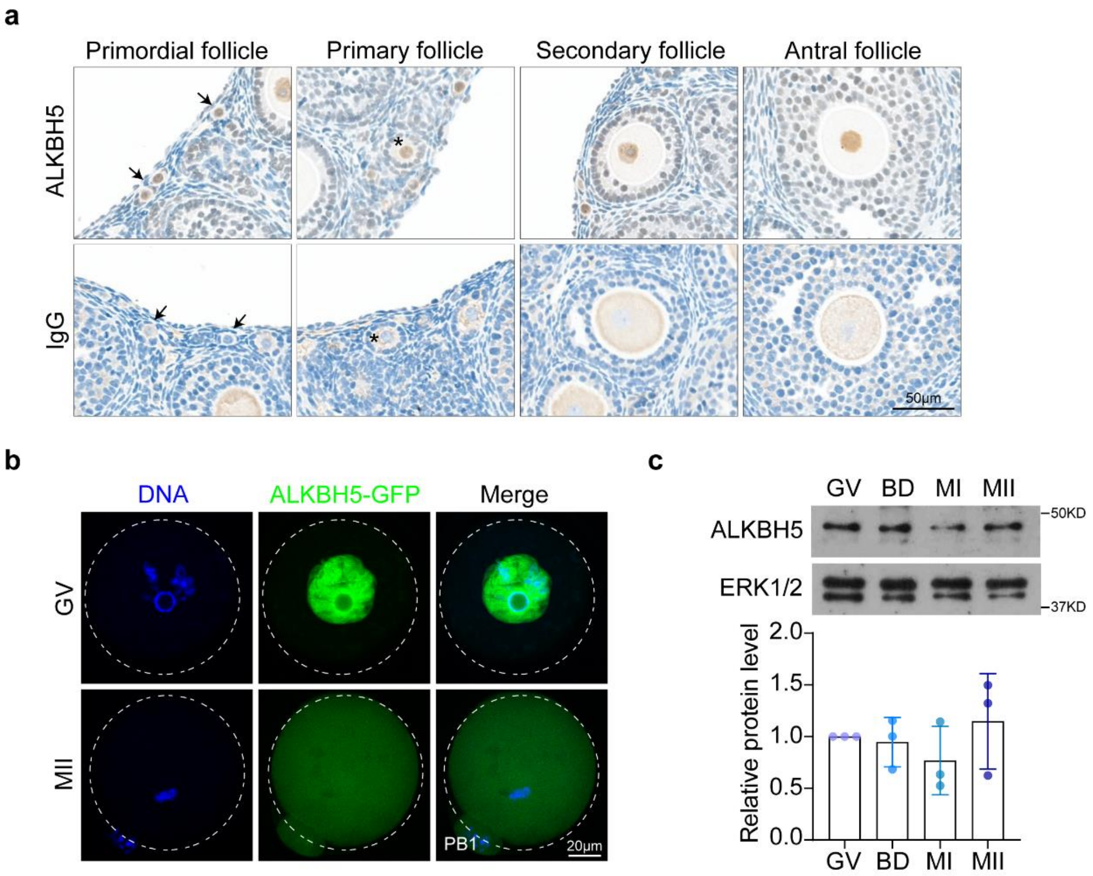

Supplementary Figure 1. Dynamic ALKBH5 distributions during meiosis in mouse oocytes. a. Immunohistochemistry results showing ALKBH5 location in oocyte during follicle development. The dark arrow indicates the primordial follicle enveloped by a layer of squamous cells and asterisk highlights the primary follicle. Immunohistochemistry images of IgG are shown as negative control. Each experiment was repeated independently for three times with similar results. **b.** Representative images showing dynamic subcellular localization of ALKBH5 in oocyte during meiotic maturation. Mouse oocytes are microinjected with *Alkbh5*-Flag-eGFP mRNA at GV stage and then maintained in M16 medium with milrinone for 6 h before being released into milrinone-free medium for *in vitro* maturation. The outliners of oocytes are marked by white dashed circle. PB1, first polar body. Each experiment was repeated independently for three times with similar results. **c.** Western blot results representing the expression patterns of ALKBH5 through meiotic maturation with quantifications of ALKBH5 at each stage (lower panel). The data in the bar graph (top) are presented as mean ± SD. ERK1/2 is applied as control. GV: 0 h, BD: 4 h, MI: 8 h, MII: 14 h (hours

in culture). n=3 biologically independent experiments. Source data are provided as a Source Data file.

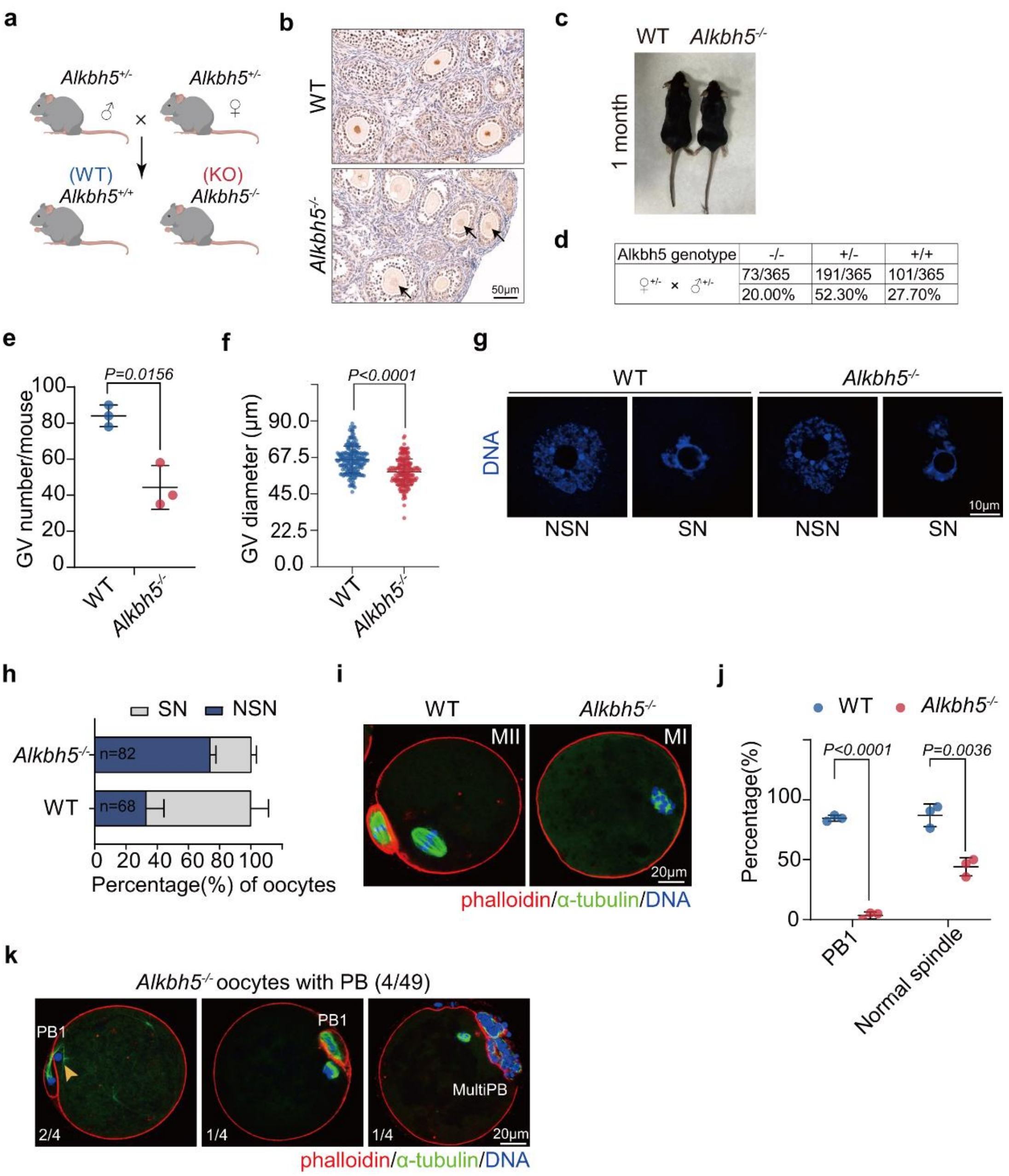

Supplementary Figure 2. Genetic knockout of *Alkbh5* and analysis of *Alkbh5*$^{-/-}$ female phonotype. a. Scheme showing intercrossing of mice heterozygous for the knockout allele to generate *Alkbh5*$^{-/-}$ mice. **b.** Immunohistochemistry results showing ALKBH5 location in WT and *Alkbh5*$^{-/-}$ovarian sections from 3-week females. Note the absence of ALKBH5 staining (black arrow) in *Alkbh5*$^{-/-}$ oocytes. Each experiment was repeated independently for three times with similar results. **c.** Representative image of WT and *Alkbh5*$^{-/-}$ mice of 1 month. Each experiment was repeated independently for three times with similar results. **d.** Table of numbers and percentages of mice per genotype retrieved from heterozygous intercrosses. n=365 animals are examined. **e.**

Number of GV oocytes derived from WT and *Alkbh5*$^{-/-}$ females following PMSG injection. The data are presented as mean ± SD. n=3 biologically independent experiments. *P* value, two-tailed Student's t test. **f.** Diameter of GV oocytes in **d**. The data are presented as mean ± SD. n=195 (WT), 189 (*Alkbh5*$^{-/-}$) oocytes are examined. *P* value, two-tailed Student's t test. *P*=3.13e-18. Source data are provided as a Source Data file. **g.** Representative images of surrounded nucleolus (SN) and non-surrounded nucleolus (NSN) GV oocytes from WT and *Alkbh5*$^{-/-}$ mice. Each experiment was repeated independently for three times with similar results. **h.** The ratio of NSN and SN oocytes in WT and *Alkbh5*$^{-/-}$ mice. Numbers of oocytes examined are indicated. **i.** Fluorescence images showing spindle and chromosome morphology in oocytes isolated from WT and *Alkbh5*$^{-/-}$ females after superovulation. Each experiment was repeated independently for three times with similar results. **j.** The percent of PB1 extrusion and normal spindle in ovulated oocytes. The data are depicted as mean ± SD over three replicates. *P*-value, two-tailed Student's t test. PB1, *P*=0.000003. **k.** Confocal microscopy of *in vitro* matured MII oocytes (16 h) from *Alkbh5*$^{-/-}$ females. Three representatives are plotted with their proportions shown on the left corner. Polar bodies are indicated. Yellow arrowhead, condensed chromatin mess. Each experiment was repeated independently for three times with similar results. Source data are provided as a Source Data file.

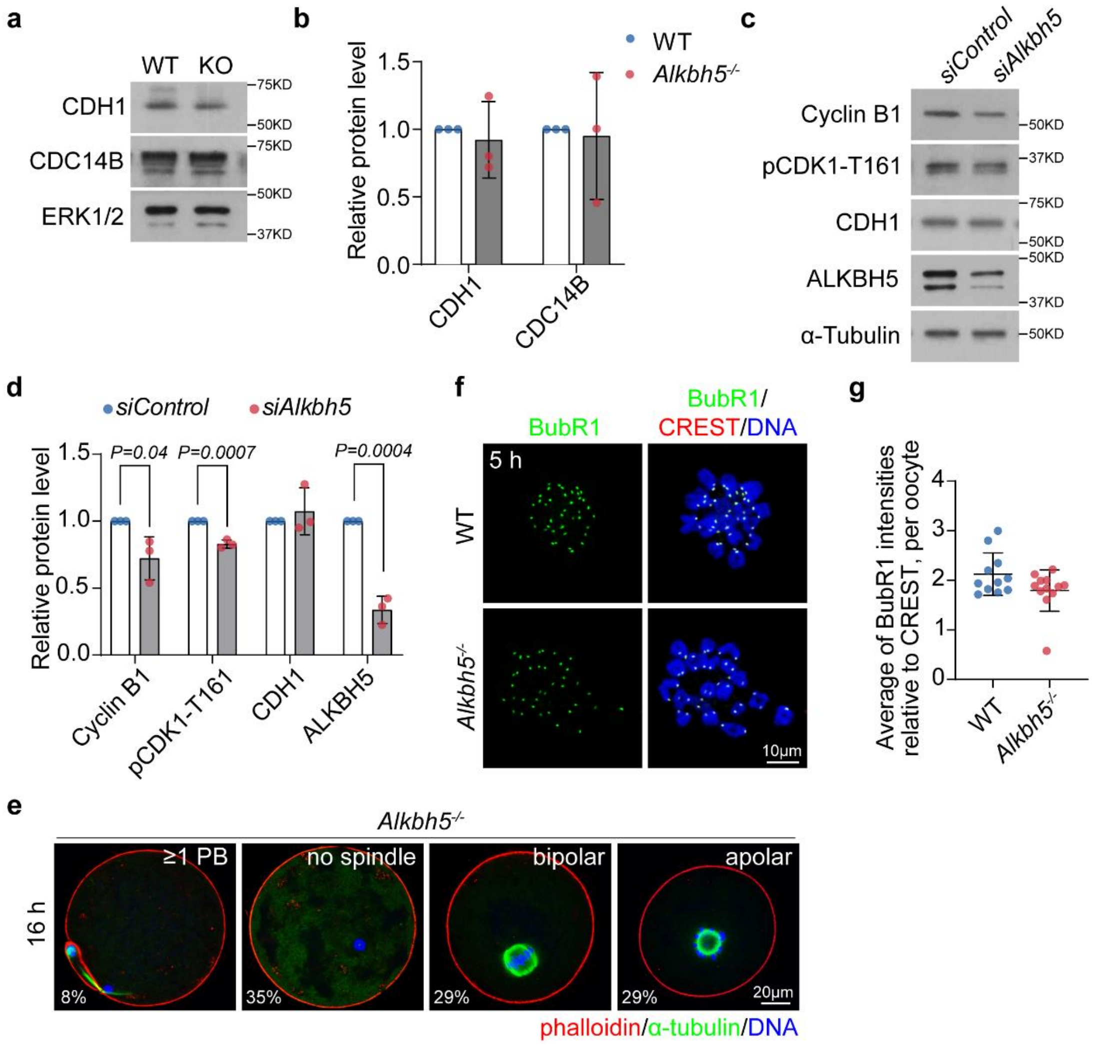

Supplementary Figure 3. *Alkbh5* depletion impairs cell cycle in meiosis and mitosis. **a-b.** Representative images (**a**) and quantifications (**b**) of MPF regulators (CDH1, CDC14B) in GVBD oocytes. ERK1/2 is referred as a loading control. The bars represent mean ± SD. n=3 biologically independent experiments. **c-d.** Representative images (**c**) and quantifications (**d**) of cell-cycle related proteins (Cyclin B1, p-CDK1 (T161), CDH1) in mouse granulosa cells interfered with control and *Alkbh5* siRNA. ERK1/2 is referred as a loading control. The bars represent mean ± SD. n=3 biologically independent experiments. *P* value, two-tailed Student's t test. **e.** Classification of spindles in *in vitro* matured *Alkbh5*$^{-/-}$ oocytes up to 16 h. Each experiment was repeated independently for three times with similar results. **f.** Immunofluorescent imaging showing SAC activation at 5 h indicated by BubR1 signals on kinetochores from both WT and *Alkbh5*$^{-/-}$ oocytes. Each experiment was repeated independently for three times

with similar results. **g.** Quantifications of BubR1 intensity on kinetochores from WT and *Alkbh5*$^{-/-}$ oocytes at 5 h. The average intensity of BubR1 per oocyte is measured as detailed in "Methods" and is normalized to Crest signal. Data is presented as mean ± SD. n=11 (WT), 12 (*Alkbh5*$^{-/-}$) oocytes are examined. Source data are provided as a Source Data file.

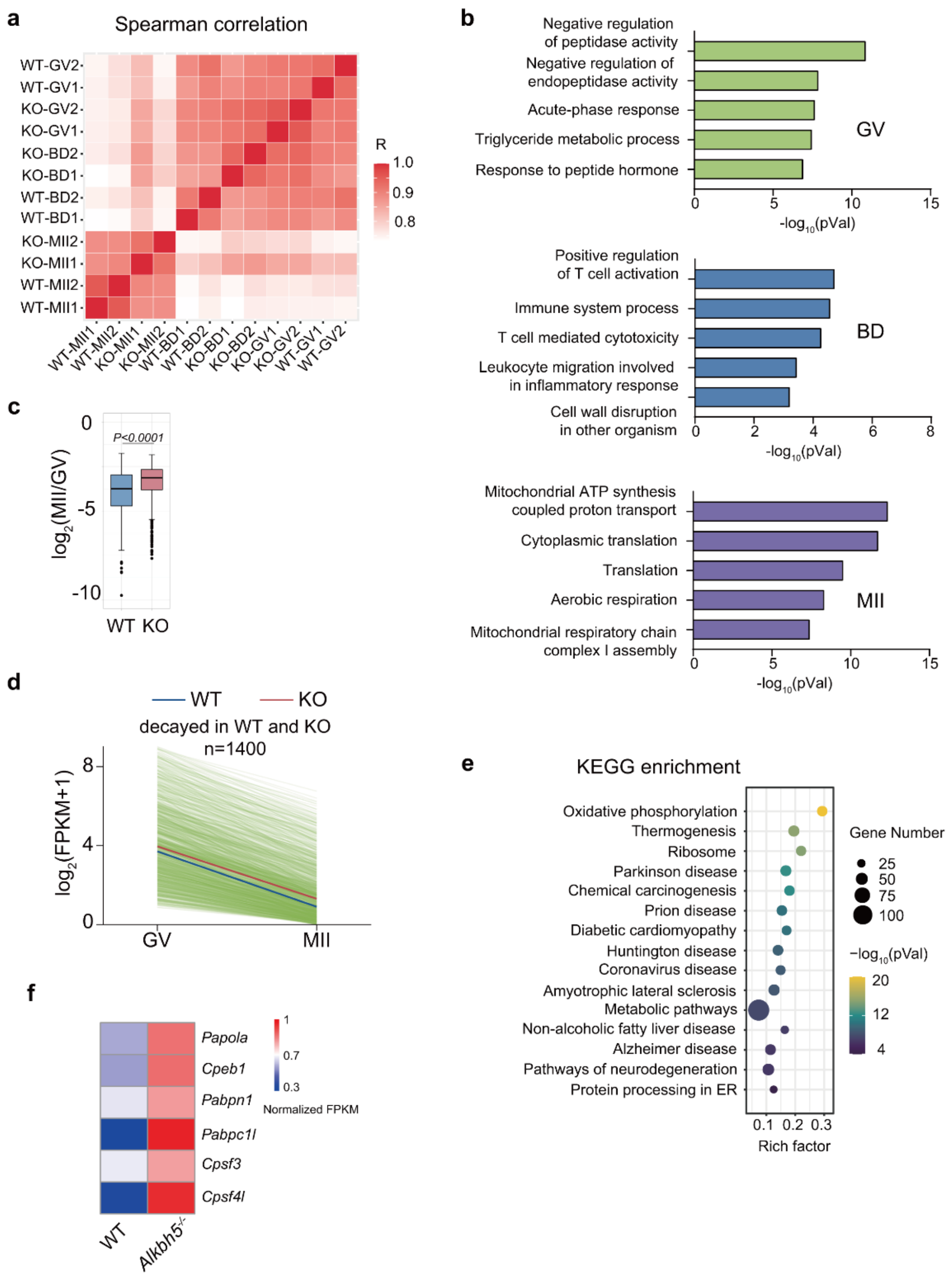

Supplementary Figure 4. Disrupted transcriptomes in oocyte maturation upon *Alkbh5* knockout. a. Correlation heatmap of examined samples at each indicated stages from WT and *Alkbh5*$^{-/-}$ mice. Data are corrected and calculated by spearman correlation analysis from two independent replicates. **b.** Gene ontology analyses of DEGs in *Alkbh5*$^{-/-}$ oocytes with respect to WT oocytes at GV, BD and MII stages respectively. Top five identified process are shown. **c**. Boxplot depicting the $\log_2$[fold change (MII/GV)] of 1400 genes that degrade in both WT and *Alkbh5*$^{-/-}$ oocytes compared between WT and KO samples. n=1400 transcripts. Data are presented as box

and whisker plots. The central line denotes median value, while the bounds of box mark the 25th to 75th percentiles and whiskers refer to minimum and max values by Tukey. *P* values, two-tailed Mann-Whitney test. *P*=4.07e-17. **d**. Degradation patterns of maternal transcripts (1400 genes) during GV-MII transition in WT and *Alkbh5*$^{-/-}$ oocytes. Each green line represents the expression level of an individual gene. The blue and red line represent the median expression levels in WT and *Alkbh5*$^{-/-}$ oocyte, respectively. **e.** Scattered graph showing KEGG enrichment on transcripts that are degraded in WT oocytes, but are stable in *Alkbh5*$^{-/-}$ oocytes during GV-MII transition. Top 15 pathways of interest are shown. **f.** Heatmap showing the expression levels of genes associated with polyadenylation in *Alkbh5*$^{-/-}$ oocytes compared to the controls. Source data are provided as a Source Data file.

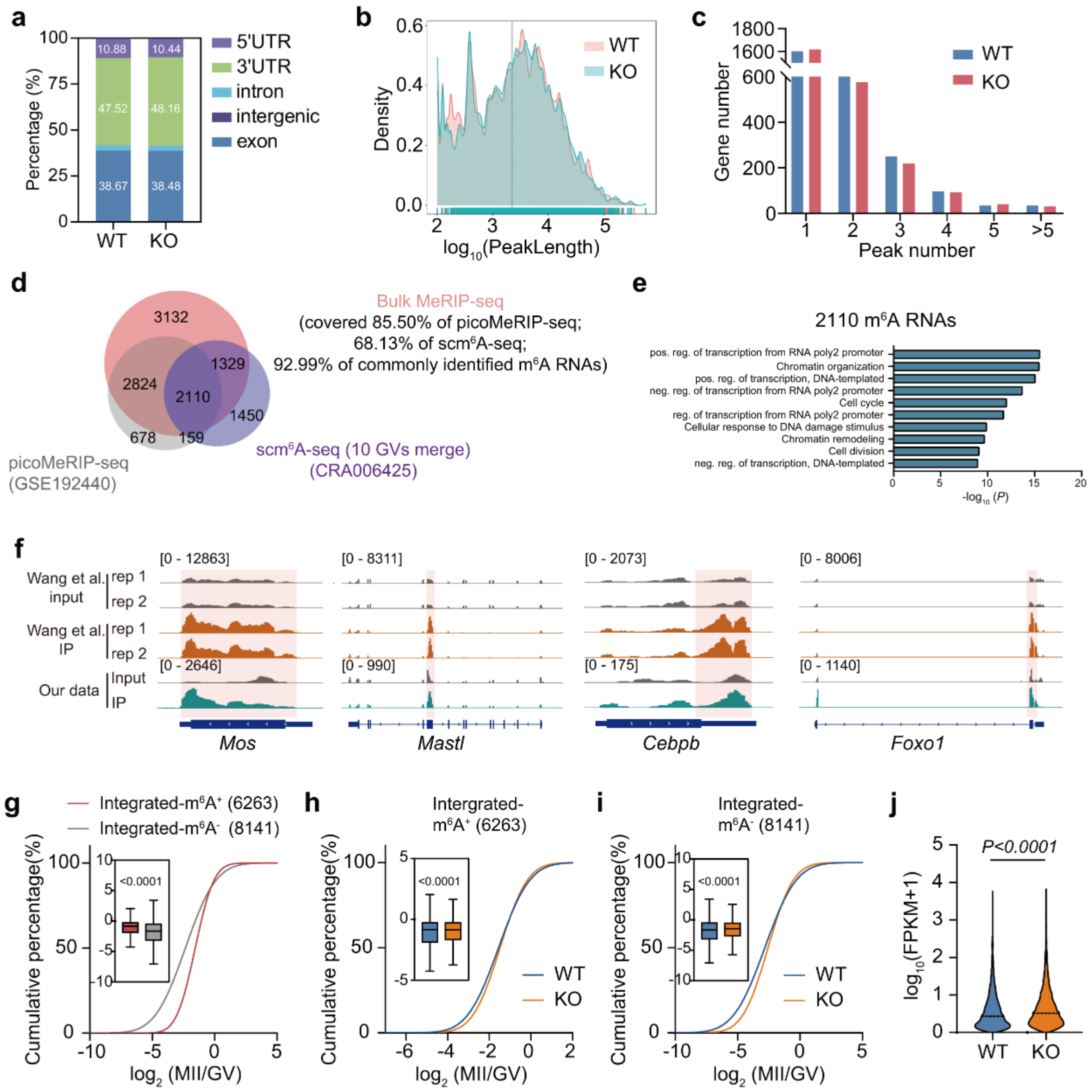

Supplementary Figure 5. Characteristics of m^{6}A modifications in *Alkbh5$^{-/-}$* oocytes. **a.** Distribution of m^{6}A peaks scanning around gene body. **b.** Density prevalence of peak length detected from WT and *Alkbh5$^{-/-}$* samples. **c.** Correlation between peak number and gene counts in WT and *Alkbh5$^{-/-}$* datasets. **d.** Venn diagram showing the overlaps of identified m^{6}A-modified RNAs in GV oocytes from picoMeRIP-seq, scm^{6}A-seq and our datasets. **e.** GO analysis of commonly identified m^{6}A-marked RNAs by different methods. pos. positive; neg. negative; reg. regulation. **f.** IGV snapshots showing m^{6}A distributions of the representative conserved m^{6}A-modified genes (*Mos*, *Mastl*, *Cebpb*, *Foxo1*) detected by different methods. The sample-specific scale ranges are indicated in brackets. **g.** Cumulative fraction and boxplot (inset) showing the difference in degradation rates (log_2[fold change (MII/GV)]) between the integrated m^{6}A-modified

and unmodified RNAs in WT oocytes. *P* values, two-tailed Mann-Whitney test. **h.** Cumulative fraction and boxplot (inset) showing the degradation rates (log_2[fold change (MII/GV)]) of the integrated m^6A-modified RNAs in WT and *Alkbh5*$^{-/-}$ oocytes, respectively. *P* values, two-tailed Mann-Whitney test. **i.** Cumulative fraction showing the degradation rates (log_2[fold change (MII/GV)]) of the integrated unmodified RNAs in WT and *Alkbh5*$^{-/-}$ oocytes, respectively. *P* values, two-tailed Mann-Whitney test. **j.** Violin plot showing the expressional levels of the integrated m^6A-modified transcripts in WT and *Alkbh5*$^{-/-}$ oocytes at MII stage. The data are presented as violin with central line denoting mean value. n=6263 transcripts. *P* values, two-tailed Mann-Whitney test. *P*=1.11e-14. Source data are provided as a Source Data file.

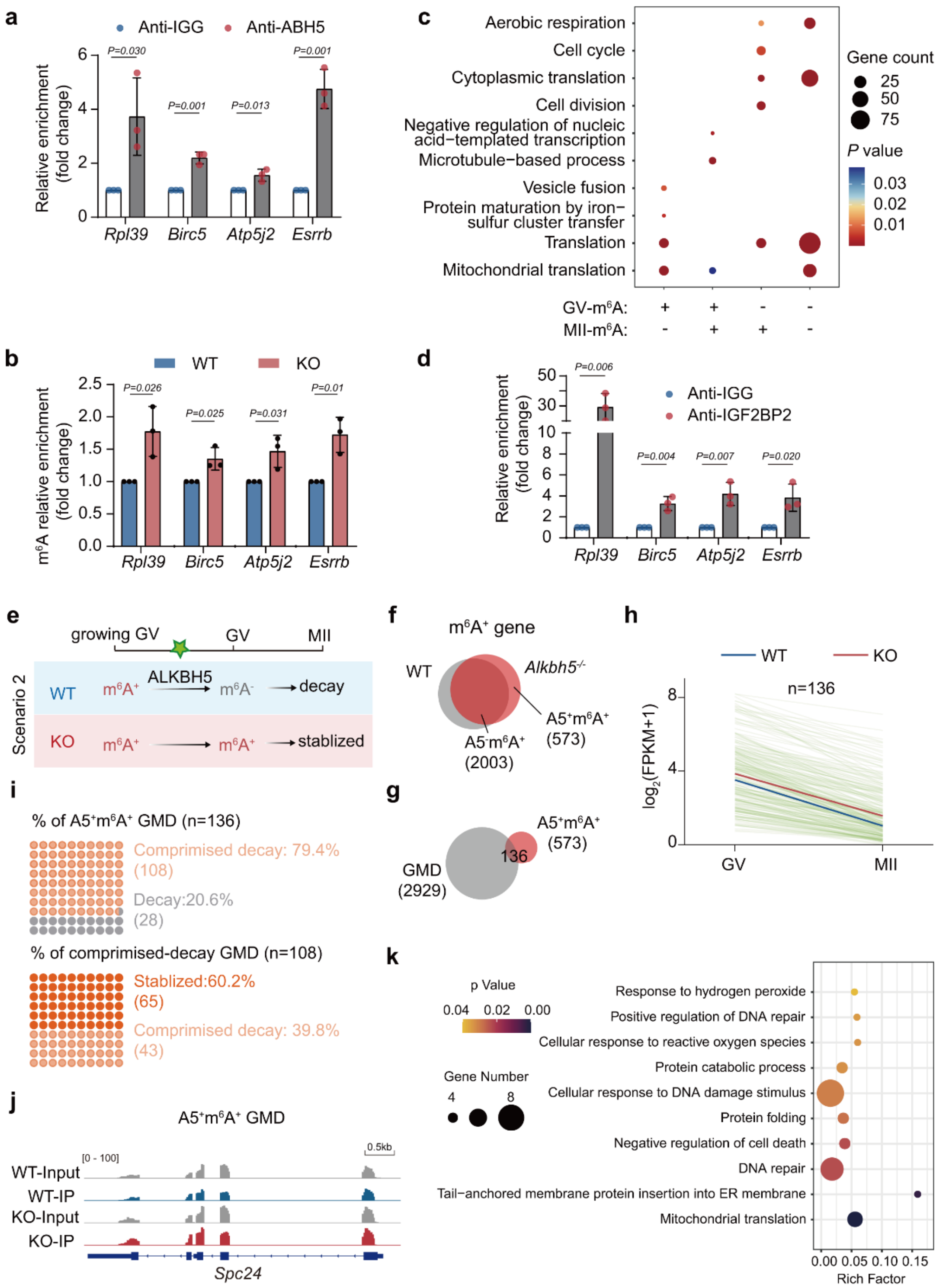

Supplementary Figure 6. ALKBH5 also functions before the occurrence of maternal RNA decay. a. RIP-qPCR analysis showing the fold changes of ALKBH5 binding ability with the indicated RNAs relative to IgG in ovarian granulosa cells from WT and *Alkbh5*$^{-/-}$ mice. The bar is mean ± SD resulting from three independent experiments. *P* value, two-tailed Student's t test. **b.** RNA immunoprecipitation results showing the relative m^{6}A enrichments among the indicated genes in mouse granulosa

cells. The data validated by qPCR are present as mean ±SD from three independent experiments and are normalized to WT GV samples. *P* value, two-tailed Student's t test. **c.** GO analyses of genes with different m^{6}A marking at GV and MII stages in GMD transcripts. **d.** RIP-qPCR analysis showing the fold changes of IGF2BP2 binding ability with the indicated RNAs relative to IgG in mouse granulosa cells. The bar is mean ± SD resulting from three independent experiments. *P* value, two-tailed Student's t test. **e.** A schematic model showing the persistent m^{6}A modification of A5$^+$m^{6}A$^+$ GMD transcripts at GV stage and repressed degradation afterwards upon *Alkbh5* depletion (ALKBH5 functions before GV stage). **f.** Venn diagram of m^{6}A-modified transcripts compared between WT and *Alkbh5*$^{-/-}$ oocytes. **g.** Venn diagram showing the overlap between A5$^+$m^{6}A$^+$ and GMD transcripts. **h.** Degradation patterns of A5$^+$m^{6}A$^+$ GMD transcripts during GV-MII transition in WT and *Alkbh5*$^{-/-}$ oocytes. Each green line represents the expression level of an individual gene. The blue and red line represent the median expression levels in WT and *Alkbh5*$^{-/-}$ oocyte, respectively. **i.** Dot plots showing percentages of repressed decayed genes in A5$^+$m^{6}A$^+$GMD transcripts (n=136) and percentages of stabilized genes in affected transcripts (n=108). **j.** IGV screenshot demonstrating m^{6}A distributions among the representative A5$^+$m^{6}A$^+$GMD gene *Spc24*. m^{6}A enrichment on WT and KO IP samples are highlighted in blue and red respectively. **k.** GO analysis of A5$^+$m^{6}A$^+$GMD transcripts. Source data are provided as a Source Data file.
