# Supplementary material for: ALKBH5 controls the meiosis-coupled mRNA clearance in oocytes by removing the N 6-methyladenosine methylation
Source: Nat Commun. 2023 Oct 17;14:6532. doi: 10.1038/s41467-023-42302-6 (PMC10582257; doi:10.1038/s41467-023-42302-6)
Supplement: Supplementary file 3 — Description of Additional Supplementary Files [file 41467_2023_42302_MOESM3_ESM.pdf]

Description of Additional Supplementary Files

File name: Supplementary Data 1
Description: DEGs in *Alkbh5*$^{-/-}$ GV oocytes

File name: Supplementary Data 2
Description: DEGs in *Alkbh5*$^{-/-}$ BD oocytes

File name: Supplementary Data 3
Description: DEGs in *Alkbh5*$^{-/-}$ MII oocytes

File name: Supplementary Data 4
Description: Gene list for destabilized genes across the GV-MII transition

File name: Supplementary Data 5
Description: Gene list for GMD genes

File name: Supplementary Data 6
Description: Gene list for m^{6}A-modified transcripts in WT and *Alkbh5*$^{-/-}$ oocytes

File name: Supplementary Data 7
Description: Primer sequences used in this study

File name: Supplementary Data 8
Description: Summary information of antibodies

File name: Supplementary Data 9
Description: Summary information of key resources

File name: Supplementary Data 10
Description: The mapping information of RNA-seq and m^{6}A meRIP-seq
