# Supplementary material for: ALKBH5 controls the meiosis-coupled mRNA clearance in oocytes by removing the N 6-methyladenosine methylation
Source: Nat Commun. 2023 Oct 17;14:6532. doi: 10.1038/s41467-023-42302-6 (PMC10582257; doi:10.1038/s41467-023-42302-6)
Supplement: Supplementary file 10 — Supplementary Data 7 [file 41467_2023_42302_MOESM10_ESM.pdf]

Supplementary Data 7: Primer sequences used in this study

Primer name	Targeted gene	Application	Sequence
WT-F	*Alkbh5*	Genotyping of WT allele	5'-GACAGCAAGGATATGGGCCAAT-3'
WT-R			5'-CCCATATTAGGCTGGCACTTCT-3'
KO-F		Genotyping of KO allele	5'-TGGATTACCACCAACACGAATGG-3'
KO-R			5'-GCTCCAGCTTCACGAGTTTGAG-3'
Rpl39-F	*Rpl39*	RT-qPCR	5'-CTTCTCCATTCCTCCGCCATC-3'
Rpl39-R			5'-CAGCTTCGTTCTCCTCCAGTGTC-3'
Rpl23a-F	*Rpl23a*	RT-qPCR	5'-TGCTATCATCAAATTCCCAC-3'
Rpl23a-R			5'-ACATACGCCTTCTTCTCTCC-3'
Rpl5-F	*Rpl5*	RT-qPCR	5'-AGCATTGACGGTCAGCCTGGTG-3'
Rpl5-R			5'-CTGACCCATGATGTGCTTCCGATG-3'
Rps13-F	*Rps13*	RT-qPCR	5'-CCCAGGTCCGTTTTGTGACT-3'
Rps13-R			5'-GTGCTTTCGGACAGCAACAG-3'
Atp5j2-F	*Atp5j2*	RT-qPCR	5'-CGAGCTGGATAATGATGCGGGA-3'
Atp5j2-R			5'-GCAGTAGCTGAAAACCACGTAGG-3'
Birc5-F	*Birc5*	RT-qPCR	5'-GAGGCTGGCTTCATCCACTG-3'
Birc5-R			5'-CTTTTTGCTTGTTGTTGGTCTCC-3'
Uchl1-F	*Uchl1*	RT-qPCR	5'-GATTAACCCCGAGATGCTGA-3'
Uchl1-R			5'-CCGATGGTACCACAGGAGTT-3'
Esrrb-F	*Esrrb*	RT-qPCR	5'-TAAAAAGCCATTGACTAAGATCGTC-3'
Esrrb-R			5'-CAATTCACAGAGAGTGGTCAGG-3'
Actb-F	*Actb*	RT-qPCR	5'-TGATCCACATCTGCTGGA-3'
Actb-R			5'-GAAGAGCTACGAGCTGCC-3'
oligo(dT)-anchor		PAT assay	5'-GCGAGCTCCGCGGCCGCGT_{12}-3'
Rpl39-P2	*Rpl39*	PAT assay	5'-CTGGTAACAAAATCAGGTACAACTCTAAG-3'
Rpl23a-P2	*Rpl23a*	PAT assay	5'-AGTCAGCCATGAAGAAAATAGAGGACAA-3'
Rpl5-P2	*Rpl5*	PAT assay	5'-TACACAGGTCAGGTAAACAGTTAAC-3'
Rps13-P2	*Rps13*	PAT assay	5'-AAAATCTTGAGAATCCTCAAGTCCAAAG-3'
Atp5j2-P2	*Atp5j2*	PAT assay	5'-AACAAGTACATCAACGTTCGGAAAG-3'
Birc5-P2	*Birc5*	PAT assay	5'-GTGGCTTTGCTCTATTGTGACGTGGACTTAAGC-3'
Uchl1-P2	*Uchl1*	PAT assay	5'-TTGGTTTGCAGCTTTAGCACTTAGAA-3'
Actb-P2	*Actb*	PAT assay	5'-AAATAAGTGGTTACAGGAAGTCCCTCAC-3'
Rpl39-m^6A-F	*Rpl39*	m^6A-RIP-qPCR	5'-GGATTCACACAATGGCAAGACTGAG-3'
Rpl39-m^6A-R			5'-AAGGGCAATTCAGTACTTTATAAACCAG-3'
Atp5j2-m^6A-F	*Atp5j2*	m^6A-RIP-qPCR	5'-AGAGGGGTCGCTGGAGG-3'
Atp5j2-			5'-AGCAGTATGTGAACCAGTCACC-3'

m^{6}A-R			
Birc5-m^{6}A-F	*Birc5*	m^{6}A-RIP-qPCR	5'-GAGGCTGGCTTCATCCACTG-3'
Birc5-m^{6}A-R			5'-CTTTTTGCTTGTTGTTGGTCTCC-3'
Esrrb-m^{6}A-F	*Esrrb*	m^{6}A-RIP-qPCR	5'-GGGCCTAGCAGGGTCAGAG-3'
Esrrb-m^{6}A-R			5'-AGATTTCAGATACATGGGACTGGATG-3'
Igf2bp2-siRNA-F	*Igf2bp2*	RNAi	5'-GAGGGCUUGACCAUAAAGATT-3'
Igf2bp2-siRNA-R			5'-UCUUUAUGGUCAAGCCCUCTT-3'
Alkbh5-ORF-F	*Alkbh5*	Plasmid reconstruction	5'-CCGCTCGAGATGGCGGCCGCCAGCGGCTA-3'
Alkbh5-ORF-R			5'-CTCTAGATCAGTGTCTCCTCATCTTCACCTTGCGGG-3'
