# Supplementary material for: ALKBH5 controls the meiosis-coupled mRNA clearance in oocytes by removing the N 6-methyladenosine methylation
Source: Nat Commun. 2023 Oct 17;14:6532. doi: 10.1038/s41467-023-42302-6 (PMC10582257; doi:10.1038/s41467-023-42302-6)
Supplement: Supplementary file 11 — Supplementary Data 8 [file 41467_2023_42302_MOESM11_ESM.pdf]

Supplementary Data 8: Summary information of antibodies

Antibodies	Dilution	Source	Identifier
Rabbit anti-ALKBH5	1:1000 for WB<br>1:200 for IHC	Sigma	Cat#HPA007196
Rabbit anti-ERK1/2	1:1000 for WB	Cell Signaling Technology	Cat#9102
Mouse anti-Cyclin B1	1:500 for WB	Santa Cruz	Cat#sc-245
Goat anti-Cyclin B2	1:1000 for WB	R&D systems	Cat#AF6004-SP
Rabbit anti-p-CDK1(T161)	1:500 for WB	Cell Signaling Technology	Cat#9114S
Mouse anti-FZR1 (CDH1)	1:1000 for WB	Abcam	Cat#ab77885
Rabbit anti-CDC14B	1:1000 for WB	Abcam	Cat#ab203675
Rabbit anti-Securin	1:1000 for WB	Abcam	Cat#ab79546
Mouse anti-CDC20	1:500 for WB	Santa Cruz	Cat#sc-13162
Mouse anti-α-Tubulin-FITC	1:1000 for immunostaining	Sigma	Cat#F2168
Rabbit anti-TPX2	1:500 for immunostaining	Abcam	Cat#ab252944
Mouse anti-Pericentrin	1:500 for immunostaining	BD transduction laboratories	Cat#611814
Alexa Fluor 594 phalloidin	1:400 for immunostaining	Invitrogen	Cat#A12381
Human anti-Crest	1:400 for immunostaining	Antibodies Incorporated	Cat#15-235-0001
Sheep anti-BubR1	1:800 for immunostaining	Abcam	Cat#ab28193
Rabbit anti-m^6A	3 ug for RIP	Synaptic Systems	Cat#202003
Rabbit anti-ALKBH5	3 ug for RIP	Proteintech	Cat#16837-1-AP
Rabbit anti-IGF2BP2	3 ug for RIP	Proteintech	Cat#11601-1-AP
Rabbit anti-IGF2BP3	3 ug for RIP	Proteintech	Cat#14642-1-AP
Rabbit anti-YTHDF2	3 ug for RIP	Proteintech	Cat#24744-1-AP
Rabbit anti-YTHDC1	3 ug for RIP	Proteintech	Cat#14392-1-AP
HRP-goat anti-mouse IgG	1:5000 for WB	Bio-Rad	Cat#1706515
HRP-goat anti-rabbit IgG	1:5000 for WB	Bio-Rad	Cat#1706515
Alexa Fluor 594 donkey anti-mouse IgG (H+L)	1:1000 for immunostaining	Thermo Scientific	Cat#R37115
Alexa Fluor 488 donkey anti-rabbit IgG (H+L)	1:1000 for immunostaining	Jackson ImmunoResearch	Cat#711-546-152
Alexa Fluor 488 donkey anti-goat IgG (H+L)	1:1000 for immunostaining	Jackson ImmunoResearch	Cat#705-546-147
