# Supplementary material for: ALKBH5 controls the meiosis-coupled mRNA clearance in oocytes by removing the N 6-methyladenosine methylation
Source: Nat Commun. 2023 Oct 17;14:6532. doi: 10.1038/s41467-023-42302-6 (PMC10582257; doi:10.1038/s41467-023-42302-6)
Supplement: Supplementary file 12 — Supplementary Data 9 [file 41467_2023_42302_MOESM12_ESM.pdf]

Supplementary Data 9: Summary information of key resources

Reagent/resource	Source	Identifier
**Bacterial strains**		
DH5α	Cowin Biotech	Cat#CW0808
**Chemicals**		
Roscovitine	Selleck	Cat#S1153
Reversine	MCE	Cat#HY-14711
Milrinone	Sigma	Cat#M4659
Triton X-100	BBI Life Science	Cat#A600198-0500
Paraformaldehyde	Aladdin	Cat#C104188
Dithiothreitol	BBI Life Science	Cat#A620058-0005
M2 medium	Sigma	Cat#M7167
M16 medium	Sigma	Cat#M7292
KSOM medium	Millipore	Cat#MR-121
Penicillin-streptomycin	Gibco	Cat#15140-122
Mineral oil	Sigma	Cat#M8410
Dulbecco's Modified Eagle's Medium	Sigma	Cat#D2906
Fetal bovine serum	Vistech	Cat#SE200-ES
GlutaMAX	Gibco	Cat#35050061
Bovine serum albumin	Sigma	Cat#A1470
lysis buffer	Cell Signaling Technology	Cat#9803
PVDF membrane	Roche	Cat#43487300
Pierce ECL Western Blotting Substrate	Thermo Scientific	Cat#32106
T4 DNA Ligase	New England biolabs	Cat#M0202T
T4 DNA Ligase	New England biolabs	Cat#M0202S
DpnI endonuclease	New England biolabs	Cat#R0176S
NotI endonuclease	New England biolabs	Cat#R0189V
XhoI endonuclease	New England biolabs	Cat#R0146V
XbaI endonuclease	New England biolabs	Cat#R0145V
DAPI staining solution	Sangon Biotech	Cat# E607303-0002
SlowFade Diamond Antifade Mountants	Invitrogen	Cat#36967
Protein A/G PLUS-Agarose	Santa Cruz	Cat#sc-2003
TRIzol	Takara	Cat#T9108
ERCC RNA Spike-In Mix	Invitrogen	Cat#4456740
dsDNA Fragmentase	New England biolabs	Cat#M0348S
**Commercial Assays**		
Lipofectamine RNAiMAX	Invitrogen	Cat#13778150
mMESSAGE SP6 kit	Invitrogen	Cat#AM1340
Poly(A) Tailing kit	Invitrogen	Cat#AM1350

MEGAclear Kit	Invitrogen	Cat#AM1980
SuperScript III First-Strand System	Invitrogen	Cat#18080051
RNeasy Mini Kit	QIAGEN	Cat#74106
Click-iT RNA Imaging Kits	Invitrogen	Cat#C10330
Click-iT HPG Alexa Fluor Protein Synthesis Assay Kits	Invitrogen	Cat#C10428
Q5® High-Fidelity DNA Polymerase amplify system	New England biolabs	Cat#M0491
One Step TB Green® PrimeScript™ RT-PCR Kit	Takara	Cat# RR066A
SMART-Seq v4 Ultra Low Input RNA Kit	Takara	Cat# 634832
**Software and Algorithms**		
GraphPad Prism	GraphPad	https://www.graphpad.com/scientificsoftware/prism/
SnapGene	N/A	http://www.snapgene.com
Fastp	N/A	https://github.com/OpenGene/fastp
HISAT2	N/A	http://daehwankimlab.github.io/hisat2
ExomePeak2	N/A	https://bioconductor.org/packages/release/bioc/html/exomePeak2.html
ANNOVAR	N/A	http://www.openbioinformatics.org/annovar/
MEME2	N/A	http://meme-suite.org
HOMER	N/A	http://homer.ucsd.edu/homer/motif
RBPsuite	N/A	http://www.csbio.sjtu.edu.cn/bioinf/RBPsuite/
IGV	PMID:21221095	https://igv.org/
DAVID	PMID:19131956	https://david.ncifcrf.gov/
Metascape	PMID:30944313	https://metascape.org/
Image J	NIH	https://imagej.nih.gov/ij/
