# Supplementary material for: ALKBH5 controls the meiosis-coupled mRNA clearance in oocytes by removing the N 6-methyladenosine methylation
Source: Nat Commun. 2023 Oct 17;14:6532. doi: 10.1038/s41467-023-42302-6 (PMC10582257; doi:10.1038/s41467-023-42302-6)
Supplement: Supplementary file 14 — Reporting Summary [file 41467_2023_42302_MOESM14_ESM.pdf]

nature portfolio

Corresponding author(s): DAPR and NCOMMS-23-16285B

Last updated by author(s): Oct 5, 2023

Reporting Summary

Nature Portfolio wishes to improve the reproducibility of the work that we publish. This form provides structure for consistency and transparency in reporting. For further information on Nature Portfolio policies, see our Editorial Policies and the Editorial Policy Checklist.

Statistics

For all statistical analyses, confirm that the following items are present in the figure legend, table legend, main text, or Methods section.

n/a	Confirmed	
☐	☒	The exact sample size (*n*) for each experimental group/condition, given as a discrete number and unit of measurement
☐	☒	A statement on whether measurements were taken from distinct samples or whether the same sample was measured repeatedly
☐	☒	The statistical test(s) used AND whether they are one- or two-sided<br>*Only common tests should be described solely by name; describe more complex techniques in the Methods section.*
☒	☐	A description of all covariates tested
☐	☒	A description of any assumptions or corrections, such as tests of normality and adjustment for multiple comparisons
☐	☒	A full description of the statistical parameters including central tendency (e.g. means) or other basic estimates (e.g. regression coefficient) AND variation (e.g. standard deviation) or associated estimates of uncertainty (e.g. confidence intervals)
☐	☒	For null hypothesis testing, the test statistic (e.g. *F*, *t*, *r*) with confidence intervals, effect sizes, degrees of freedom and *P* value noted<br>*Give P values as exact values whenever suitable.*
☒	☐	For Bayesian analysis, information on the choice of priors and Markov chain Monte Carlo settings
☐	☒	For hierarchical and complex designs, identification of the appropriate level for tests and full reporting of outcomes
☐	☒	Estimates of effect sizes (e.g. Cohen's *d*, Pearson's *r*), indicating how they were calculated

Our web collection on statistics for biologists contains articles on many of the points above.

Software and code

Policy information about availability of computer code

Data collection	Illumina Novasecr 6000 system (PE150). ABI QuantStudio5 Real-Time PCR system. Leica Application Suite X. Eppendorf micromanipulation System.
Data analysis	For analysis of RNA-seq and m6A-seq data, poor quality reads were trimmed using Cutadapt (version 2.10). Valid data were then aligned to mouse reference genome (GRCm38/mm10) by Hisat2 (version 2.0.5) and only uniquely mapped sequence sorted by SAMtools (version 1.6) were subjected to the downstream analysis. Differential expression analysis was accomplished using DESeq2. Functional annotation was complemented using DAIVD website and Metascape. Peak calling and differential peak analysis were performed with R package exomePeak2 followed by annotation of m6A peaks (IP/input>2, p<0.05) using ANNOVAR. De novo motif enrichment was accomplished with appliance of MEME2 and HOMER (version 4.11.1).<br>Images were collected using ImageJ 1.53a Java 1.8.0_112 software (64 bit).<br>For all other data analysis, GraphPad Prism 8 was applied.

For manuscripts utilizing custom algorithms or software that are central to the research but not yet described in published literature, software must be made available to editors and reviewers. We strongly encourage code deposition in a community repository (e.g. GitHub). See the Nature Portfolio guidelines for submitting code & software for further information.

Data

Policy information about availability of data

All manuscripts must include a data availability statement. This statement should provide the following information, where applicable:

- Accession codes, unique identifiers, or web links for publicly available datasets
- A description of any restrictions on data availability
- For clinical datasets or third party data, please ensure that the statement adheres to our policy

All raw sequencing data of RNA-seq and m6A-seq in this study have been deposited at NCBI Gene Expression Omnibus (GEO) under accession codes: GSE229771 and GSE229774, respectively. The complete m6A-seq and RNA-seq data generated in this study are summarized in Supplementary Data 1-6 and 10. Source data are provided with this paper.

Research involving human participants, their data, or biological material

Policy information about studies with human participants or human data. See also policy information about sex, gender (identity/presentation), and sexual orientation and race, ethnicity and racism.

Reporting on sex and gender	It is not relevant to this study.
Reporting on race, ethnicity, or other socially relevant groupings	It is not relevant to this study.
Population characteristics	It is not relevant to this study.
Recruitment	It is not relevant to this study.
Ethics oversight	It is not relevant to this study.

Note that full information on the approval of the study protocol must also be provided in the manuscript.

Field-specific reporting

Please select the one below that is the best fit for your research. If you are not sure, read the appropriate sections before making your selection.

☒ Life sciences ☐ Behavioural & social sciences ☐ Ecological, evolutionary & environmental sciences

For a reference copy of the document with all sections, see nature.com/documents/nr-reporting-summary-flat.pdf

Life sciences study design

All studies must disclose on these points even when the disclosure is negative.

Sample size	No statistical methods were used to predetermine sample size, and no sample-size calculations were performed. Sample sizes in this study were determined based on the previous studies in this field, availability of Alkbh5 KO samples and technical feasibility.<br>For fertility test, three WT female mice and three Alkbh5-/- female mice were used.<br>Analysis of oocyte developmental competence in vitro was performed in three independent sets of samples with size varying from 35 to 90 oocytes.<br>Microinjection of oocytes with siRNA was complemented on seven independent sets of samples with size varying between 11 and 25 oocytes.<br>RT-qPCR was performed in independent sets of samples (5-10 oocytes/ each) [1].<br>Western blot was performed in independent sets of samples (100-200 oocytes/ each lane) [2].<br>Analysis of poly(A) tail lengths by PCR was conducted using total RNA isolated from 200 oocytes for each sample [1].<br>For RNA-seq, 10 oocytes of each stage were collected [3].<br>For m6A-seq, approximately 2800 GV oocytes of corresponding genotypes were collected.<br>The sample sizes of other experiments are indicated in figure legends and methods with the detection of statistical significance to support the conclusions.<br>[1] Qian-Qian Sha et al. CNOT6L couples the selective degradation of maternal transcripts to meiotic cell cycle progression in mouse oocyte. EMBO J. 2018, 37(24):e99333.<br>[2] Chao Yu et al. CRL4-DCAF1 ubiquitin E3 ligase directs protein phosphatase 2A degradation to control oocyte meiotic maturation. Nat Commun. 2015, 6:8017<br>[3] Yue Hu et al. Oocyte competence is maintained by m6A methyltransferase KIAA1429-mediated RNA metabolism during mouse follicular development. Cell Death Differ. 2020, 27(8):2468-2483.
Data exclusions	In most studies, data were not excluded from the analyses. In analyses of RNA-seq and m6A-seq dataset, genes with low expression levels (FPKM 1 in all samples) were excluded, as they were regarded as unexpressed genes. This exclusion criteria was pre-established and used in the previous studies (PMID: 30478191, 33004802).
Replication	All the experiments in this study have been replicated for three times or more, except for PAT assay, RNA-seq and m6A-seq. PAT assay was

Replication | performed once due to the limited availability of Alkbh5 KO MII samples. RNA-seq of oocytes at each stage was repeated twice. m6A-seq of GV oocytes from WT and Alkbh5-/- female mice was performed once due to the limited availability of Alkbh5 KO oocytes. All other attempts at replication were successful.

Randomization | All WT and Alkbh5-/- oocytes from multiple mice were randomly allocated into the different experimental groups.

Blinding | No blinding experiments were performed, as the same investigator set up the group allocation during data collection and analyzed the results

Reporting for specific materials, systems and methods

We require information from authors about some types of materials, experimental systems and methods used in many studies. Here, indicate whether each material, system or method listed is relevant to your study. If you are not sure if a list item applies to your research, read the appropriate section before selecting a response.

Materials & experimental systems

n/a	Involved in the study
☐	☒ Antibodies
☒	☐ Eukaryotic cell lines
☒	☐ Palaeontology and archaeology
☐	☒ Animals and other organisms
☒	☐ Clinical data
☒	☐ Dual use research of concern
☒	☐ Plants

Methods

n/a	Involved in the study
☒	☐ ChIP-seq
☒	☐ Flow cytometry
☒	☐ MRI-based neuroimaging

Antibodies

Antibodies used

All antibodies used in this study are listed in Table S2.
Rabbit anti-ALKBH5 (1:1000 for WB, 1:200 for IHC, Sigma, Cat#HPA007196)
Rabbit anti-ERK1/2 (1:1000 for WB, Cell Signaling Technology, Cat#9102)
Mouse anti-Cyclin B1 (1:500 for WB, Santa Cruz, Cat#sc-245)
Goat anti-Cyclin B2 (1:1000 for WB, R&D systems, Cat#AF6004-SP)
Rabbit anti-p-CDK1 (T161) (1:500 for WB, Cell Signaling Technology, Cat#91145)
Mouse anti-FZR1 (CDH1) (1:1000 for WB, Abcam, Cat#ab77885)
Rabbit anti-CDC14B (1:1000 for WB, Abcam, Cat#ab203675)
Rabbit anti-Securin (1:1000 for WB, Abcam, Cat#ab79546)
Mouse anti-CDC20 (1:500 for WB, Santa Cruz, Cat#sc-13162)
Mouse anti-α-Tubulin-FITC (1:1000 for immunostaining, Sigma, Cat#F2168)
Rabbit anti-TPX2 (1:500 for immunostaining, Abcam, Cat#ab252944)
Mouse anti-Pericentrin (1:500 for immunostaining, BD transduction laboratories, Cat#611814)
Alexa Fluor 594 phalloidin (1:400 for immunostaining, Invitrogen, Cat#A12381)
Human anti-Crest (1:400 for immunostaining, Antibodies Incorporated, Cat#15-235-0001)
Sheep anti-BubR1 (1:800 for immunostaining, Abcam, Cat#ab28193)
Rabbit anti-m6A (for RIP, Synaptic Systems, Cat#202003)
Rabbit anti-ALKBH5 (3 ug for RIP, Proteintech, Cat#16837-1-AP)
Rabbit anti-IGF2BP2 (3 ug for RIP, Proteintech, Cat#11601-1-AP)
Rabbit anti-IGF2BP3 (3 ug for RIP, Proteintech, Cat#14642-1-AP)
Rabbit anti- YTHDF2 (3 ug for RIP, Proteintech, Cat#24744-1-AP)
Rabbit anti- YTHDC1 (3 ug for RIP, Proteintech, Cat#14392-1-AP)
HRP-goat anti-mouse IgG (1:5000, Bio-Rad, Cat#1706515)
HRP-goat anti-rabbit IgG (1:5000, Bio-Rad, Cat#1706515)
Alexa Fluor 594 donkey anti-mouse IgG (H+L) (1:1000, Thermo Scientific, Cat#R37115)
Alexa Fluor 488 donkey anti-rabbit IgG (H+L) (1:1000, Jackson ImmunoResearch, Cat#711-546-152)
Alexa Fluor 488 donkey anti-goat IgG (H+L) (1:1000, Jackson ImmunoResearch, Cat#705-546-147)

Validation

Rabbit anti-ALKBH5
https://www.sigmaaldrich.cn/CN/zh/product/sigma/hpa007196
Validated in previous study (PMID: 24489119).
Rabbit anti-ERK1/2
https://www.cellsignal.cn/products/primary-antibodies/p44-42-mapk-erk1-2-antibody/9102?site-searchtype=Products&N=4294956287&Ntt=9102&fromPage=plp&_requestid=2313746
Validated in previous study (PMID: 37012400)
Mouse anti-Cyclin B1
https://www.scbt.com/p/cyclin-b1-antibody-gns1?requestFrom=search
Validated in previous study (PMID: 1310257)
Goat anti-Cyclin B2
https://www.rndsystems.com/cn/products/human-mouse-cyclin-b2-antibody_af6004
Validated in previous study (PMID: 34559563)
Rabbit anti-p-CDK1 (T161)
https://www.cellsignal.cn/products/primary-antibodies/phospho-cdc2-thr161-antibody/9114?site-searchtype=

Products&N=4294956287&Ntt=9114s&fromPage=plp&_requestid=2315952
Validated in previous study (PMID: 36159636)
Mouse anti-FZR1 (CDH1)
https://www.abcam.cn/products/primary-antibodies/fzr1-antibody-ar382-ab77885.html
Validated in previous study (PMID: 20223764)
Rabbit anti-CDC14B
https://www.abcam.cn/products/primary-antibodies/cdc14b-antibody-ab203675.html
Validated in previous study (PMID: 32328643)
Rabbit anti-Securin
https://www.abcam.cn/products/primary-antibodies/securin-antibody-epr3240-ab79546.html
Validated in previous study (PMID: 36450246)
Mouse anti-CDC2O
https://www.scbt.com/p/cdc20-antibody-e-7
Validated in previous study (PMID: 10679238)
Mouse anti-α-Tubulin-FITC
https://www.sigmaaldrich.cn/CN/zh/product/sigma/f2168
Validated in previous study (PMID: 20814902)
Rabbit anti-TPX2
https://www.abcam.cn/products/primary-antibodies/tpx2-antibody-epr23182-47-ab252944.html
Validated in previous study (PMID: 33907841)
Mouse anti-Pericentrin
https://www.bdbiosciences.com/zh-cn/products/reagents/microscopy-imaging-reagents/immunofluorescence-reagents/purifiedmouse-anti-mouse-pericentrin.611814
Validated in previous study (PMID: 30478191)
Alexa Fluor 594 phalloidin
https://www.thermofisher.cn/order/catalog/product/A12381?SID=srch-hj-A12381
Validated in previous study (PMID: 30478191)
Human anti-Crest
https://www.antibodiesinc.com/products/anti-centromere-protein-antibody-15-234
Validated in previous study (PMID: 36260995)
Sheep anti-BubR1
https://www.abcam.cn/products/primary-antibodies/bubr1-antibody-ab28193.html
Validated in previous study (PMID: 33553181)
Rabbit anti-m6A
https://www.sysy.com/product/202003#list
Validated in previous study (PMID: 34480159)
Rabbit anti-ALKBH5
https://www.ptgcn.com/products/ALKBH5-Antibody-16837-1-AP.htm
Validated in previous study (PMID: 33020597)
Rabbit anti-IGF2BP2
https://www.ptgcn.com/products/IGF2BP2-Antibody-11601-1-AP.htm
Validated in previous study (PMID: 35606490)
Rabbit anti-IGF2BP3
https://www.ptgcn.com/products/IGF2BP3-Antibody-14642-1-AP.htm
Validated in previous study (PMID: 25117712)
Rabbit anti-YTHDF2
https://www.ptgcn.com/products/YTHDF2-Antibody-24744-1-AP.htm
Validated in previous study (PMID: 33442060)
Rabbit anti-YTHDC1
https://www.ptgcn.com/products/YTHDC1-Antibody-14392-1-AP.htm
Validated in previous study (PMID: 36441764)
HRP-goat anti-mouse IgG
https://www.bio-rad.com/zh-cn/sku/1706516-goat-anti-mouse igg h I hrp conjugate?ID=1706516
HRP-goat anti-rabbit IgG
https://www.bio-rad.com/zh-cn/sku/1706515-goat-anti-rabbit igg h I hrp conjugate?ID=1706515
Alexa Fluor 594 donkey anti-mouse IgG (H+L)
https://www.thermofisher.cn/cn/zh/antibody/product/Donkey-anti-Mouse IgG H L Secondary-Antibody-Polyclonal/R37115
Alexa Fluor 488 donkey anti-rabbit IgG (H+L)
https://www.jacksonimmuno.com/catalog/products/711-546-152
Alexa Fluor 488 donkey anti-goat IgG (H+L)
https://www.jacksonimmuno.com/catalog/products/705-546-147

Animals and other research organisms

Policy information about studies involving animals; ARRIVE guidelines recommended for reporting animal research, and Sex and Gender in Research

Laboratory animals

The experimental mice were all of C57BL/6 genetic background, Alkbh5+/-mice were designed and purchased from Gem Pharmatech. Animals were maintained under a specific pathogen-free (SPF) facility with an appropriately controlled environment (12 h light /12 h dark cycle with temperature of18-23 ?) and humidity (50-70%) and with easy access to food and water. Female mice of 4-6 weeks were used for oocyte collection. For mating test, Alkbh5+/- female mice (2-month-old) was mated with WT C57BL/6 male mice (2-month-old).

Wild animals	No wild animals were used in this study.
Reporting on sex	Female mice were used for oocyte collection and further analysis.
Field-collected samples	No field-collected samples were used in this study.
Ethics oversight	All animal experiments were approved by local animal ethnical committee and were strictly conducted in compliance with the Animal Care and Use Committee of Zhejiang University (File no. ZJU20220520).

Note that full information on the approval of the study protocol must also be provided in the manuscript.
